# The effect of unexpected rewards on decision making in cuttlefish

**DOI:** 10.1038/s41598-022-06443-w

**Published:** 2022-02-15

**Authors:** Tzu-Ting Chung, Anne-Sophie Darmaillacq, Ludovic Dickel, Chuan-Chin Chiao

**Affiliations:** 1 Institute of Molecular Medicine, National Tsing Hua University, Hsinchu, Taiwan, ROC; 2 Ethologie Animale et Humaine (EthoS)-University of Caen Normandie, Université de Rennes I, CNRS, Rennes, France; 3 Institute of Systems Neuroscience, National Tsing Hua University, Hsinchu, Taiwan, ROC; 4 Department of Life Science, National Tsing Hua University, Hsinchu, Taiwan, ROC

**Keywords:** Animal behaviour, Emotion

## Abstract

Despite numerous studies demonstrating the cognitive ability of cephalopods, there is currently no study showing an emotion-like behavior in this group of animals. To examine whether cuttlefish have different internal states, we developed a behavioral paradigm to assess if prior surprised events are able to alter the choice made by cuttlefish. By presenting unexpected food rewards to cuttlefish before the test, we investigated whether the reaction time of choosing between two shrimps, an intuitive response toward the prey without previous learning, at three different levels of discriminative tests (easy, difficult, and ambiguous), are different compared to the one without an unexpected reward. This behavioral paradigm serves to demonstrate whether cuttlefish are aware of ambiguous situations, and their choice outcome and reaction time are dependent of their internal states. The results show that the response latency was significantly shortened in the difficult and ambiguous tests when choosing from two shrimps that are either moderately different in size or similar sizes, respectively, when cuttlefish have received unexpected rewards before the test. These results were compared with tests during which the cuttlefish did not receive any reward in advance. Furthermore, this shortening of latency did not result in a difference in choice outcome during the difficult and ambiguous tests. Interestingly, even when cuttlefish have obtained the expected food rewards or simply made tentacular strike without prey capture each time before test, these prior experiences were sufficient to shorten the response latency in the difficult and ambiguous tests. However, different from the result of unexpected rewards, food consumption alone or prey capture failure did affect the choice outcome during the simple and difficult tests. Taken together, our findings suggest that pre-test treatments of unexpected and expected rewards or simply unsuccessful visual attack seem to induce cuttlefish to adopt different foraging behaviors. This context dependent decision making suggests that cuttlefish’s foraging strategies are influenced by the previously surprised event and their internal states. It also shows a speed-accuracy tradeoff in difficult and ambiguous situations when foraging for prey. This observation may lead to a future investigation of the presence of emotional state in cephalopods.

## Introduction

Emotions are salient and short-lived responses to specific classes of environmental situations. It assists organisms when confronting with various problems posed by the environment. From the evolutionary perspectives, emotion enables animals to adequately integrate the external and internal information, rapidly respond to threats, and seek for valuable resources. These adaptive values provided by emotion may increase survival and reproduction^1–5^. The neural and hormonal mechanisms underlying the emotion appear in vertebrates^6^. Some homolog mechanisms are also found in invertebrates^4^. It seems that the genetically hardwired emotions appear to be highly conserved across a wide phylogenetic range. This suggests that the key features of emotion may be organized in systems which preserved in ancient origins and went through a ubiquitous selection. Thus, the principle derived from the research in model organisms which investigates the evolutionary origins and neurobiological underpinnings of emotion could be generalized across phylogeny.

An animal’s emotional-states can be characterized by three essential elements: a subjective experience, a physiological response, and a behavioral or expressive response^2,7,8^. One of the major obstacles in confirming whether animals other than humans experience the internal emotion-state is the difficulty of determining the nature of an animal’s subjective experience. Comparing with studies involving humans, who are able to verbally express subjective feelings regarding the emotional state, studies involving non-human animals usually adopt non-verbal techniques in order to assess the conscious experiencing of emotion. Researchers tend to focus on the behavioral, neurophysiological, and cognitive components of emotions^9^. In fact, Darwin proposed from a functional and evolutionary standpoint that emotional expression can be recognized by expressive behaviors, not only in humans, but also in some mammalian species such as primates^10^. These affective responses hence point to the evidence of the common evolutionary ancestry of humans and other animals. Therefore, by combining all the components, we might be able to grasp the emotional states experienced by an individual.

Based on the approaches outlined above, numerous studies on animal emotion-like state have been published on a range of vertebrates, including fish, chickens, mice, pigs, and dolphins^11–15^. Not until recently have these methods been adapted to investigate the emotion-like state of invertebrates^16–19^. Among the various methodologies available, the judgment bias test has been the most frequently used cognitive approach for assessing the emotion-like state of animals. Emotional states influence the processes of cognition and cognitive processes are closely link to the elicitation of an emotion internally^20,21^. Judgment bias thus is regarded as subject’s interpretation of ambiguous information which is affected by internal emotion-like state^22,23^. In other words, the judgment bias task enables us to assess the interaction between emotional states and cognitive interpretations of ambiguous situations. In the standard judgment bias test, the subjects first learn to discriminate a positive cue associated with a reward (CS+) from another negative cue associated with no reward or punishment (CS−) in the training phase. Once the subjects can readily distinguish between the positive and negative stimuli, it is followed by a series of manipulation of stimulus. In the testing phase, successive discrimination tests that involve various ambiguous stimuli which lie between the positive and negative cues are presented to the subjects. The hypothesis is that subjects that tend to display a high expectation of reward in presence of ambiguous information are interpreted as showing an optimistic behavior, an indication of positive emotional state. In contrast, subjects that tend to display a higher expectation of punishment or lower expectation of reward in responding to ambiguous stimuli are interpreted as showing a pessimistic behavior, an indication of negative emotional state^24–26^.

Cephalopods possess elaborate brains and are equipped with advanced cognitive abilities^27,28^. They are endowed with a sophisticated nervous system that supports them by exhibiting strikingly flexible behavioral repertoires. These include problem solving and tool use^29,30^, anti-predatory behaviors^31–33^ and social behaviors^34,35^. All of them are evidence of the evolution of cephalopod intelligence^36^. Furthermore, a few studies were conducted to investigate whether cephalopods have the affective pain experience in the past decade^37–39^. A more recent study revealed that octopuses exhibit nociception specific behaviors which provide the evidence supporting the existence of a pain state in cephalopods^40^. It should be noted that the aforementioned cognitive abilities and intelligence are not limited to cephalopods only. In fact, there are some evidence supporting that arthropods, particularly bees and ants, also have behavioral flexibility and advanced cognitive abilities^41,42^.

In cuttlefish, we have shown that when animals make a decision between one large shrimp and two small shrimps, this depends on their appetite state; that is, they prefer a single larger shrimp when they are starving, but two smaller shrimps when they are satiated^43^. In a separate study, we have also shown that foraging decision-making by cuttlefish is dependent on the relative values learned from previous experience^44^. Cuttlefish typically preferred the larger quantity in the one vs. two shrimp test. However, after cuttlefish were primed under conditions where they were given a small reward for choosing one shrimp in a zero vs*.* one test, they then chose one shrimp significantly more frequently in the following one vs*.* two test.

Despite the fact that cephalopods have these elaborated cognitive abilities, up to the present, there has not been any studies demonstrating that these mollusks have emotion-like behavior. In the present study, we developed a behavioral paradigm inspired by the aforementioned judgment bias test to investigate the emotion-like state of cuttlefish. Differing from the standard judgement bias test^45^, this behavioral paradigm has no training phase and relies on animal’s intuitive preying behavior with a simultaneous (not successive) discrimination task. This simplified protocol is easy to operate and their choice of different preys represents an instinct response independent of learning. We took advantage of the food preference of cuttlefish for larger shrimp when there is a choice between larger and smaller prey^43^. Three different level discrimination tests (easy, difficult, and ambiguous) which comprise two different size shrimps were used in this behavioral test. The assumption is that, by presenting three different level discrimination tests, the cuttlefish that has received an unexpected reward would be more optimistic in the ambiguous situation. Animals that had not received an unexpected reward would not make the ambiguous choice as speedily. Thus, an increase in optimism may imply that the cuttlefish have an altered affective state after receiving an unexpected reward.

## Materials and methods

### Subjects

The eggs of pharaoh cuttlefish (*Sepia pharaonis*), which had been spawned by wild-caught females, were reared by the Aquatic Biotech Company Ltd. (Yilan, Taiwan) during February 2020 and 2021. They were then transported to National Tsing Hua University (Hsinchu, Taiwan). The animals were reared further in the laboratory using two closed recirculating aquaculture systems (700 L each) that were maintained at approximately a temperature of 24 ℃ and a salinity of 33 parts per thousand. The photoperiod of the recirculating aquaculture systems was a 12/12 h light/dark cycle. One month after hatching, the juvenile cuttlefish were housed individually in porous containers floating inside the rearing tank. Depending on their mantle length, different containers were used (ML < 2 cm, kept in a container 16 cm × 11 cm × 6 cm; ML > 2 cm, kept in a container 24 cm × 16 cm × 6 cm). Outside of the testing period, they were fed a diet of prey items ad libitum consisting of post-larvae white shrimp (*Litopenaeus vannamei*) and freshwater shrimp (*Neocaridina denticulate*) of suitable size, the size being decided based on the mantle length of each cuttlefish. The experiments were conducted when the cuttlefish were 2-month-old (ML ~ 2 cm). Cuttlefish of the same age were introduced as a replacement if an individual died during the experiments. In total, 26 cuttlefish were used in the present study. All procedures were approved by the Institutional Animal Care and Use Committee of the National Tsing Hua University (Protocol # 10911H005).

### Experimental apparatus and procedure

**Figure 1:**
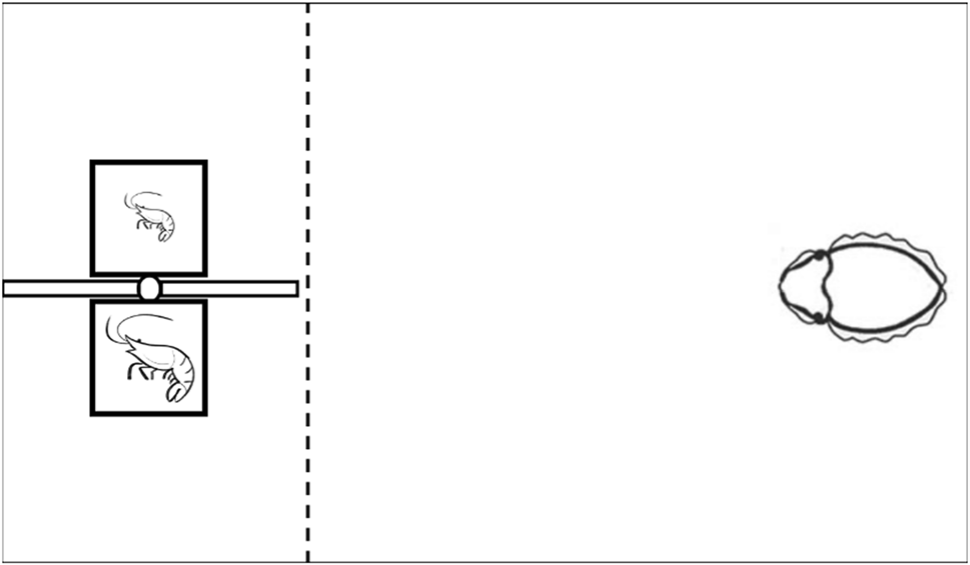
Schematic representation of the top view of the experimental set-up. The two chambers containing shrimps are presented in front of the cuttlefish, and the animal is motivated to swim toward one of the two chambers. The imaginary dotted line indicates the decision point, where the cuttlefish determines the choice.

The experimental apparatus consisted of a two-chamber device made up of two small transparent plastic boxes (2.5 cm × 2.5 cm × 2.5 cm) separated by a protruding plastic sheet designed to force the cuttlefish to make an irrevocable choice (the two-alternative forced-choice design, or 2AFC; Fig. 1). Depending on the test, different sizes of shrimps were placed in two chambers and the apparatus was lowered into the floating container at the opposite side from the cuttlefish’s location. The experiment was initiated by allowing the cuttlefish to swim toward the two chambers and make a choice. Cuttlefish actively prey on the live shrimps. They capture them by shooting out two tentacles to make a strike. This behavior is visually driven in cuttlefish^46^. Once the tentacles had passed an imaginary dotted line (Fig. 1), the apparatus was lifted out of the water to reduce as much as possible the occurrence of passive avoidance learning, an inhibition of predatory behavior observed during the “prawn-in-the-tube” training procedure^47–50^. The latency from the time that cuttlefish converged their eyes on the apparatus to the time that their tentacles crossed the imaginary dotted line was recorded and called the reaction latency. The choice that cuttlefish made in each test was also recorded. To avoid reinforcement of each animal’s decision when making a choice, cuttlefish were never rewarded with any shrimp in the chamber once a choice was made. The Experiment 1 (see below) followed a within-subject design with two conditions (Control and Treatment I conditions); these conditions underwent three tests each (easy, difficult, and ambiguous). There was a total of six different tests during Experiment 1. Each cuttlefish was examined ten times during each test, thus each cuttlefish underwent a total of 60 trials, unless stated otherwise. All of the trials and tests in the Experiment 1 were presented in a random order. In the Experiments 2 and 3, two additional conditions (Treatment II and III conditions) were designed to test the alternative hypotheses (see below). Each condition also underwent three tests (easy, difficult, and ambiguous), and each cuttlefish was examined eight times during each test, thus each cuttlefish underwent a total of 24 trials, unless stated otherwise. During all the experiments, the two chambers were swapped left-and-right sequentially to minimize the cuttlefish’s visual lateralization effect^51^. Note that there were no more than six trials a day for one cuttlefish. The inter-trial-interval was at least 5 min. However, if the previous trials were in the Treatment I condition of Experiment 1, the inter-trial-interval was at least 1 h. After each cuttlefish had completed all of the trials for that day, they were fed an adequate amount of food in the absence of the apparatus. To ensure that shrimps were vigorously active during the experiment, the chambers were refreshed every 10–15 min and shaken before being lowered into the container. The tops of the chambers were marked clearly with the sizes of shrimps inside, and a digital camera (Panasonic DC-GH5S) with a 15 mm lens (Panasonic H-X015) was mounted above to record the responses of the cuttlefish. Two white LED strips (5 W) were suspended on the sides of the floating basket to provide even illumination during the experiments. All experiments were conducted in the home tank of the animals during daytime (9 a.m.–9 p.m.).

### Experimental design

#### Experiment 1: Control condition and Treatment I condition

In the Control condition, to investigate whether difficulty of discrimination affects the cuttlefish’s choice latency and outcome, three different levels of discrimination tests (easy, difficult, and ambiguous) were presented. The easy test consisted of a choice between two shrimps with a distinct different size (0.5 vs. 1), where 1 is equal to the mantle of the cuttlefish and 0.5 means that the shrimp length was a half of the large shrimp. The difficult test was a choice between two shrimps with a moderate size difference (0.75 vs. 1), where 0.75 means that the shrimp length was 75% of the large shrimp. The ambiguous test was a choice between two shrimps with a similar size (1 vs. 1).

In the Treatment I condition, to evaluate how the unexpected reward influences the cuttlefish’s choice latency and outcome during the same set of discrimination tests (easy, difficult, and ambiguous), a tiny shrimp (roughly 20% of the cuttlefish’s body length) was given to the cuttlefish before each trial started. Note that the tiny shrimp was too small to satiate the cuttlefish in the trial. Since all the trials in both conditions (Control and Treatment I) were conducted in a random order, the tiny shrimp fed before each trial in the Treatment I condition was regarded as an unexpected reward for the cuttlefish. If cuttlefish have emotion-like states, we would expect that the choice latency in the Treatment I condition should be shorter than that in the Control condition only in the ambiguous test, but not in the easy and difficult tests. This is because cuttlefish received unexpected rewards would be more optimistic in the ambiguous situation and make the choice more speedily. Furthermore, it is expected that the choice outcome (i.e., the tendency of choosing the larger shrimp) would not be affected by receiving unexpected rewards in all three tests, because the emotion-like state only alters the speed but not the accuracy in the ambiguous situation. Nine cuttlefish were used in six tests of both conditions, and each test comprises ten trials. Each animal went through at least eight trials per test (see “Supplementary material” for details).

#### Experiment 2: Treatment II condition

To investigate whether the pre-test rewards in the Treatment I condition could affect cuttlefish’s appetite during the experiment, the same size of tiny shrimp (roughly 20% of the cuttlefish’s body length) was given to the cuttlefish before each trial. Thus, this condition was regarded as expected rewards. Due to the time constraint and the cuttlefish availability, the result of Treatment II condition was compared with that of the Control in Experiment 1, rather than a new set of control that is independent and not intermixed with Treatment I trials in Experiment 1. If cuttlefish’s emotion-like states could be altered by the unexpected reward in Experiment 1, we would expect that simply increasing appetite by the expected reward in the Treatment II condition should not shorten the choice latency and change the choice tendency in the ambiguous test. Six cuttlefish were used in the Treatment II condition. This condition consists of three tests (easy, difficult, and ambiguous; as in the Experiment 1), and each of three tests comprises 8 trials. Each cuttlefish went through at least 7 trials per test (see “Supplementary material” for details).

#### Experiment 3: Treatment III condition

To determine whether the tentacle strike before capturing the tiny shrimp in the Treatment I could increase the preying motivation of cuttlefish and thus the speed of their preying behavior, the Treatment III condition was designed. In this condition, a shrimp was present to the cuttlefish and a transparent plastic sheet was placed between shrimp and cuttlefish. This configuration makes the shrimp observable but not capturable by the cuttlefish. After cuttlefish made visual attack and their tentacles stroke on the plastic sheet, cuttlefish were subjected to three levels of discrimination tests (easy, difficult, and ambiguous; as in the Experiments 1 and 2). If cuttlefish’s emotion-like states could be altered by the unexpected reward in Experiment 1, we would expect that simply elevating preying motivation by evoking tentacle strike in the Treatment III condition should not shorten the choice latency and change the choice tendency in the ambiguous test. Six cuttlefish were used in the Treatment III condition. Each of three tests in this condition comprises 8 trials. Each cuttlefish went through at least 7 trials per test (see “Supplementary material” for details).

### Data analysis

Although the sample size was not established based on a power analysis before the start of the study, it meets the minimal number of samples for a meaningful statistical analysis. Due to the availability of cuttlefish and the time limit, nine animals were used for the Experiment 1, and six animals were used for the Experiments 2 and 3. The reaction latency was scored subsequently through the analysis of videos by one of the authors (TTC) who was not blind to the conditions. To ensure that the latencies scored were reliable, a naïve person who was not related to this experiment analyzed a subset of the randomly selected videos. The Pearson correlation coefficient of these two sets of measurements was 0.94 (p < 0.01), supporting that the scores obtained from two independent scorers were highly correlated.

In the Experiment 1, since cuttlefish were used repeatedly in the Control and Treatment I conditions, the reaction latencies of each choice were compared using the non-parametric Friedman test with post hoc comparisons of the Wilcoxon Signed Ranks test and Bonferroni correction in each condition. For comparing the reaction latencies between two conditions, the Wilcoxon Signed Ranks test was used in three difficulty levels of discrimination tests. In the Experiments 2 and 3, since cuttlefish in the Control and Treatments II/III conditions were from different cohorts, the reaction latencies of cuttlefish in each test were compared using the Mann–Whitney U test. The choice outcomes of cuttlefish in each test were assessed using the non-parametric Wilcoxon Signed Ranks test. Specifically, a one-sample Wilcoxon Signed Rank test was applied to compare the proportion of choosing the larger shrimp against the chance level (50%) in each test. Data points from some trials that differ significantly from other observations were considered as outliers (two standard deviations above or below the mean), and they were excluded from the analysis (see “Supplementary material” for details). However, even if we included all outliers in the analysis, the statistical results were not affected. All statistical analyses were conducted using SPSS.

### Ethics

This work was carried out in accordance with the EU-Directive 2010/63/EU, and all procedures were approved by the Institutional Animal Care and Use Committee of the National Tsing Hua University (Protocol # 10911H005). In addition, the study is reported in accordance with ARRIVE guidelines.

## Results

**Figure 2:**
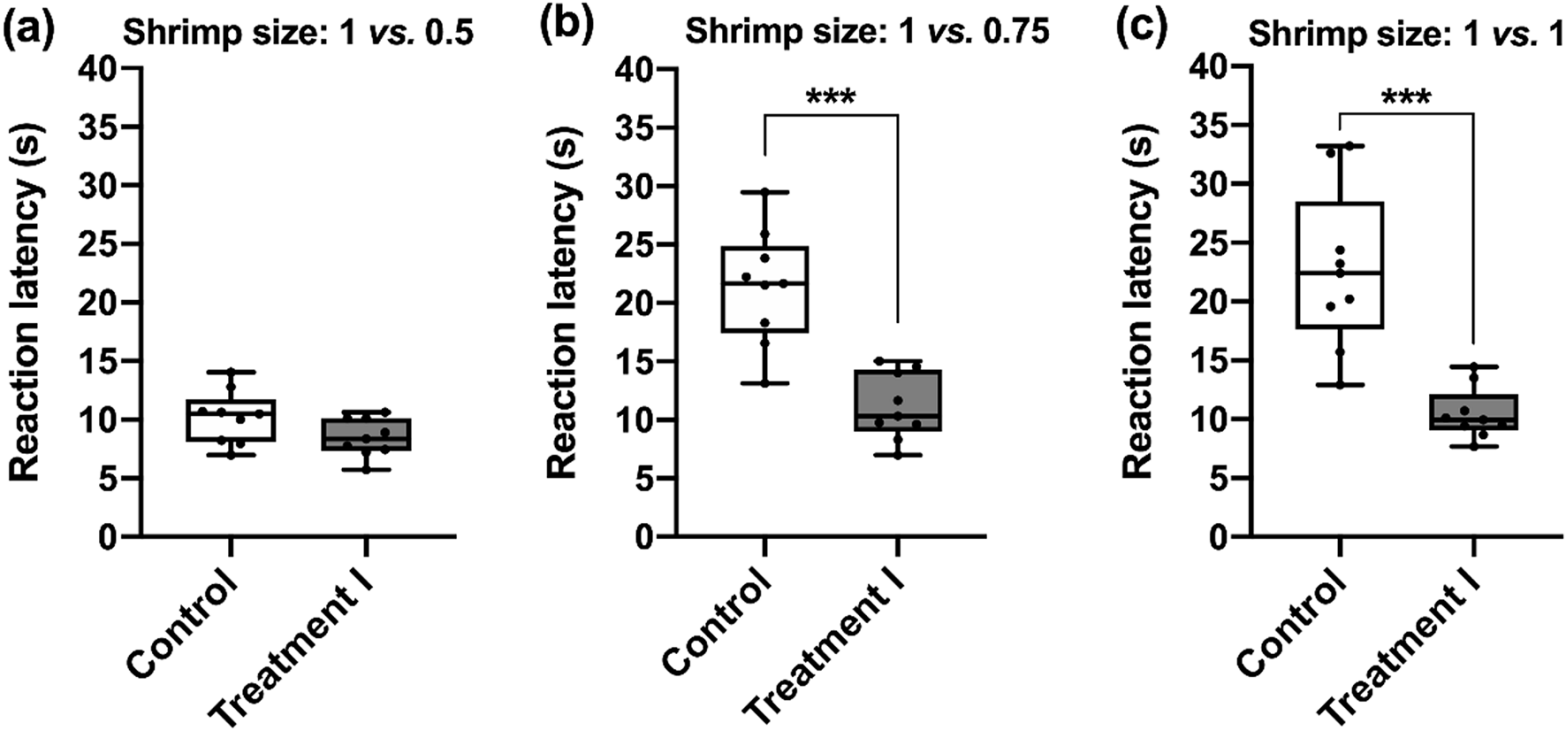
The unexpected reward decreases the reaction latency of cuttlefish making difficult and ambiguous decisions. Cuttlefish chose between two shrimps that are significantly different in size, moderately different in size, or similar in size during the easy, difficult, and ambiguous tests, respectively. (**a**) The reaction latencies were not significantly different between the two conditions during the size discrimination test involving a 1 vs*.* 0.5 decision (*p* = 0.091). (**b**) In the 1 vs*.* 0.75 test, the reaction latencies were significantly different between the cuttlefish that had received the unexpected reward and the cuttlefish that had not received the unexpected reward (*p* < 0.001). (**c**) When the shrimp size was 1 vs*.* 1, cuttlefish with the unexpected reward took significantly less time to make the decision (*p* < 0.001). *n* = 9. Error bars are s.e.m. ***p < 0.001.

In the Experiment 1, we examined if the unexpected reward was able to alter the response of the cuttlefish during an ambiguous discrimination test. In the Treatment I condition, there was no statistically significant difference in the reaction latency among three difficulty levels of discrimination tests (χ^2^(2) = 2.667, *p* = 0.264). In contrast, if we compared the reaction latencies between the Control and Treatment I conditions for three difficulty levels of discrimination tests using Wilcoxon Signed Ranks test, the results showed that the reaction latency of cuttlefish in the easy trials (1 vs. 0.5) was unaffected by the unexpected reward (*Z* = 1.688, *p* = 0.091; Fig. 2a). However, when the shrimp size was the same (1 vs*.* 1), the reaction latency of the unexpected reward-received cuttlefish was significantly decreased (*Z* = 6.344, *p* < 0.001; Fig. 2c). This result is in line with the previous study in bumblebees where the pre-test unexpected reward caused positive judgment bias in the ambiguous situation^52^. Interestingly, the unexpected reward also dramatically reduced the time that cuttlefish spent in making the decision between shrimp size 1 vs. 0.75 (*Z* = 5.608, *p* < 0.001; Fig. 2b). This indicates that the unexpected reward also was able to influence the speed of decision-making in cuttlefish. On the other hand, the reaction latency of the cuttlefish in the Control condition was proportionally correlated with the difficulty levels of discrimination tests. There was a statistically significant difference in the reaction latency among three difficulty levels of discrimination tests (χ^2^(2) = 13.556, *p* = 0.001). Post hoc analysis with Wilcoxon signed-rank tests was conducted with a Bonferroni correction applied, resulting in a significance level set at *p* < 0.017. There were statistically significant increases in the reaction latency of easy vs*.* difficult test (1 vs*.* 0.5 and 1 vs*.* 0.75, *Z* = 6.591, *p* < 0.001) and easy vs*.* ambiguous test (1 vs*.* 0. 5 and 1 vs*.* 1, *Z* = 6.912, *p* < 0.001). However, there was no significant difference between difficult and ambiguous test (1 vs*.* 0.75 and 1 vs*.* 1, Z = 0.657, *p* = 0.511). This further supports the hypothesis that the unexpected reward exerts a much stronger effect on latency when cuttlefish are making decisions during both the difficult and ambiguous discrimination tests.

**Figure 3:**
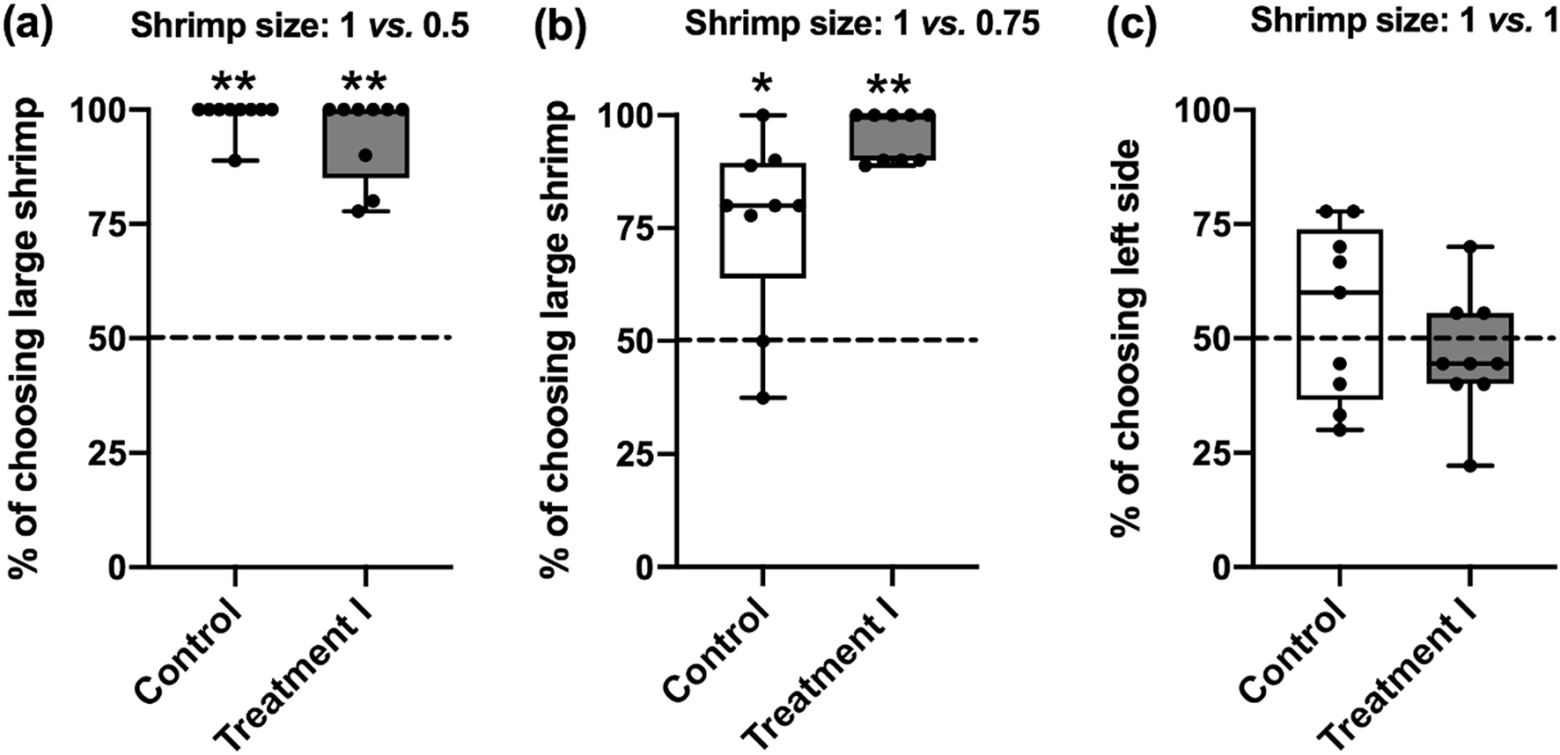
Cuttlefish can accurately discriminate two different sizes of shrimps in both the easy and difficult tests with or without the unexpected reward. (**a**) Cuttlefish tended to choose the larger shrimp when the shrimp size was 1 vs*.* 0.5 under both conditions. (**b**) When the discrimination level was 1 vs*.* 0.75, cuttlefish still preferred larger prey size in both conditions. (**c**) In the ambiguous test, cuttlefish did not show any preference in a particular side. *n* = 9. Error bars are s.e.m. *p < 0.05, **p < 0.01.

To examine the speed and accuracy trade-off in both Control and Treatment I conditions, the choice outcomes were evaluated for all six tests using a one-sample Wilcoxon Signed Rank test (Fig. 3). It is apparent that the cuttlefish in both conditions chose the larger shrimp more often than the smaller shrimp in the 1 vs*.* 0.5 test (Control condition, *Z* = 2.701, *p* = 0.007; Treatment I condition, *Z* = 2.716, *p* = 0.007; Fig. 3a) and the 1 vs*.* 0.75 test (Control condition, *Z* = 2.342, *p* = 0.019; Treatment I condition, *Z* = 2.807, *p* = 0.005; Fig. 3b). However, in contrast, they showed no preference in the 1 vs*.* 1 test (Control condition, *Z* = 0.426, *p* = 0.67; Treatment I condition, *Z* = 1.403, *p* = 0.161; Fig. 3c). This indicates that cuttlefish are able to accurately distinguish between two different sized shrimps even when they are making a fast decision about a difficult choice.

**Figure 4:**
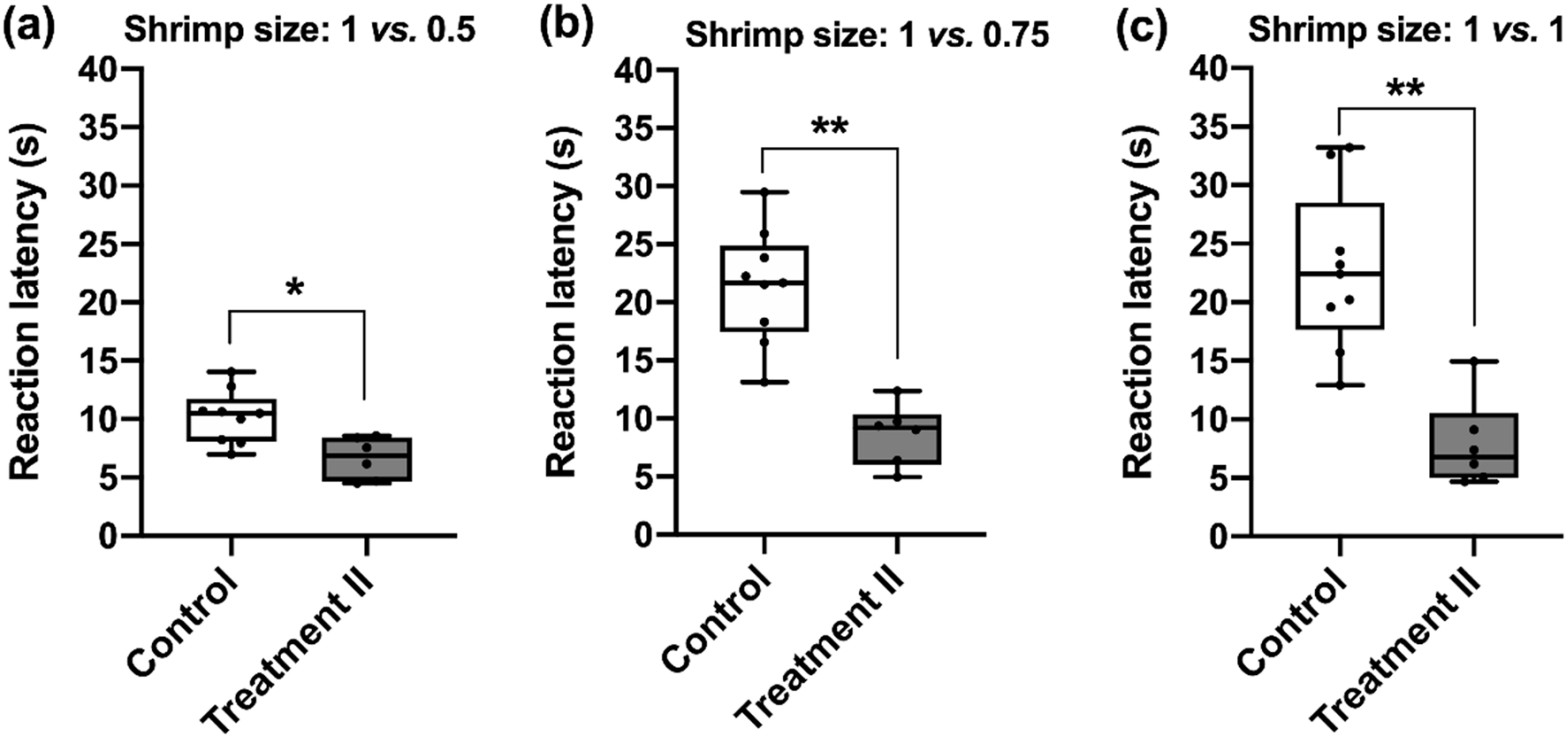
The expected reward before test decreases the reaction latency of cuttlefish in decision-making in all three levels of discrimination tests. (**a**) The reaction latencies in the 1 vs*.* 0.5 test were significantly different between the two conditions (*p* = 0.018). (**b**) In the 1 vs*.* 0.75 test, the reaction latencies of cuttlefish in the Treatment II condition was much shorter than the ones in the Control condition (*p* = 0.001). (**c**) When the shrimp size was 1 vs*.* 1, cuttlefish with the expected reward took significantly less time in making the decision (*p* = 0.002). *n* = 6. Error bars are s.e.m. *p < 0.05, **p < 0.01.

In the Experiment 2, we sought to examine the influence of the pre-test reward on the cuttlefish’s appetite. Surprisingly, it is evident that the reaction latencies of the reward-received cuttlefish decreased significantly in all three discrimination tests using the Mann–Whitney U test (1 vs*.* 0.5, *U* = 2.357, *p* = 0.018, Fig. 4a; 1 vs*.* 0.75, *U* = 3.182, *p* = 0.001, Fig. 4b; 1 vs*.* 1, *U* = 3.064, *p* = 0.002; Fig. 4c). The result thus suggests that feeding the tiny shrimp as a known reward before the test may have a significant effect on the appetite of cuttlefish, and this in turn would speed up their choice of prey.

**Figure 5:**
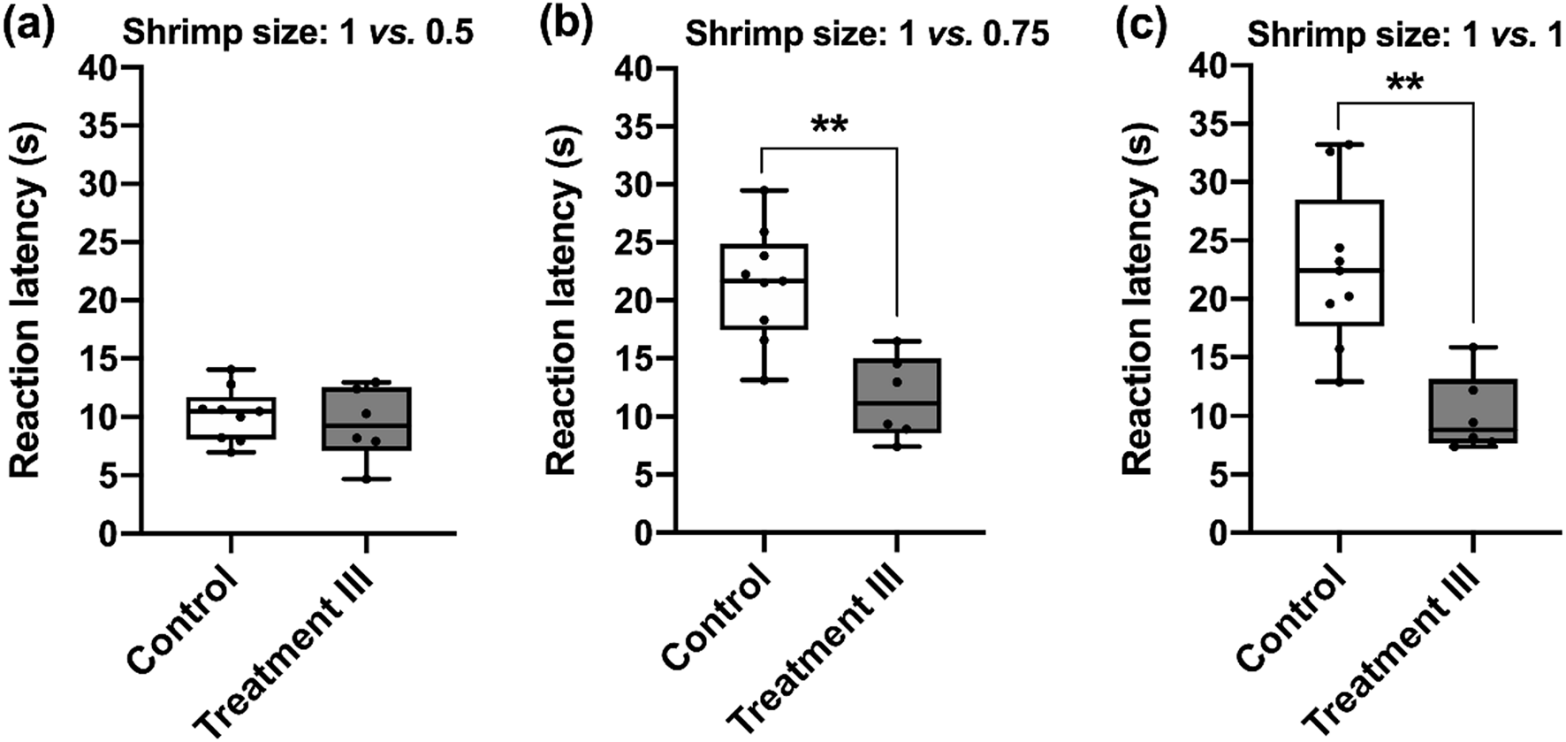
The tentacle strike alone before test decreases the reaction latency of cuttlefish making difficult and ambiguous decisions. (**a**) The reaction latencies were not significantly different between the two conditions during the 1 vs*.* 0.5 test (*p* = 0.556). (**b**) Cuttlefish with tentacle strike before test showed a significant latency reduction in the 1 vs*.* 0.75 test (*p* = 0.003). (**c**) When the shrimp size was 1 vs*.* 1, cuttlefish in the Treatment III condition had much faster reaction time in making the decision (*p* = 0.003). *n* = 6. Error bars are s.e.m. **p < 0.01.

In the Experiment 3, we went one step further to investigate whether the tentacle strike alone without eating the shrimp before the test could enhance the motivation of preying behavior in cuttlefish. The result showed that the latencies of decision-making for the easy discrimination test in this treatment condition did not differ from the one in the Control condition using the Mann–Whitney U test (*U* = 0.589, *p* = 0.556; Fig. 5a). However, cuttlefish with tentacle strike alone apparently spent less time in making decision during both difficult and ambiguous discrimination tests when compared with the animals in the Control condition (*U* = 2.946, *p* = 0.003; Fig. 5b; *U* = 2.946, *p* = 0.003; Fig. 5c). This implies that simply making the tentacle strike before test could increase the internal drive of preying behavior, and this in turn would speed up their choice of prey in both difficult and ambiguous tests.

**Figure 6:**
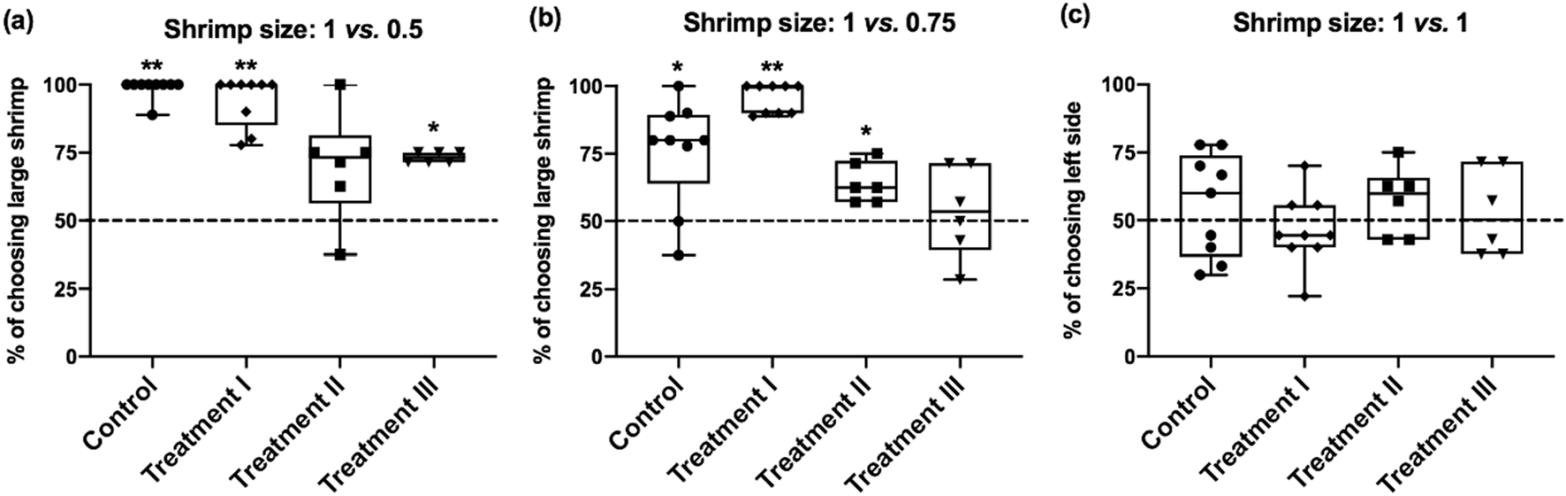
The choice preference of larger shrimp is altered when cuttlefish obtaining expected rewards or making tentacle strike before test. (**a**) Cuttlefish tended to choose the larger shrimp when the shrimp size was 1 vs*.* 0.5, except the animals from the Treatment II condition. Note that the tendency of choosing larger shrimps was also decreased in cuttlefish from the Treatment III condition. (**b**) In the 1 vs*.* 0.75 test, only the cuttlefish with tentacle strike before test had no preference for larger shrimps, though the tendency was also decreased in cuttlefish from the Treatment II condition (**c**) When the shrimp size was the same, cuttlefish did not have their preference in a particular side in all conditions. *n* = 9 for Control and Treatment I; *n* = 6 for Treatments II and III. Error bars are s.e.m. *p < 0.05, **p < 0.01.

While the results from the Experiments 2 and 3 seemed to suggest that the observed latency reduction of the cuttlefish receiving the unexpected rewards in both difficult and ambiguous discrimination tests was not due to the positive emotion-like state (Fig. 2), the choice outcomes of cuttlefish in the Experiments 2 and 3 were different from the result in the Experiment 1. It is evident that the proportion of choosing larger shrimp in the easy discrimination test was decreased in cuttlefish from the Treatments II and III conditions when compared with the animals from the Control and Treatment I conditions (Fig. 6a). Particularly, cuttlefish with the expected reward before test showed no preference on larger shrimps in the 1 vs*.* 0.5 test using a one-sample Wilcoxon Signed Rank test (Treatment II, *Z* = 1.897, *p* = 0.058). Similarly, the proportion of choosing larger shrimp in the difficult discrimination test was also decreased in cuttlefish from the Treatments II and III conditions (Fig. 6b), and the cuttlefish with tentacle strike alone before test had no preference on larger shrimps in the 1 vs. 0.75 test (Treatment III,* Z* = 0.552, *p* = 0.581). Finally, cuttlefish from all treatments and control conditions had no effect on their prey choice in the 1 vs*.* 1 test (Fig. 6c). These results suggest that the accuracy of cuttlefish in distinguishing between two different sizes of shrimps was compromised in the Treatment II and III conditions, though their speed of decision-making was increased by these treatments.

## Discussion

In the present study, we aimed to investigate whether cuttlefish are able to experience an internal emotion-like state by using a behavioral paradigm inspired by the judgment bias test to examine their choice of responses when faced with an ambiguous stimulus. In Experiment 1, it was observed that cuttlefish spent significantly more time making decisions during the ambiguous discrimination test when they did not receive an unexpected reward before the test (Fig. 2c). However, after having received the unanticipated reward, the cuttlefish took significantly less time making a choice during the same ambiguous situation. This shortened reaction latency might be an indication of a positive judgment bias in cuttlefish.

In the classical judgment bias test, the reaction latency is a vital measurement in the test^45^. Nevertheless, to obtain this response, it takes time to train subjects to associate one rewarding cue with a positive event and one unrewarding cue with a negative event before presenting the ambiguous discrimination tests. Compared with these judgement bias paradigms, it should be emphasized that, in the present study, the reaction latency of cuttlefish during the discrimination tests is a more intuitive (i.e., spontaneous or unlearned) response that is exhibited by subjects without previous learning. Thus, the results from the present experimental design may not be comparable with those of the classical judgment bias test.

Surprisingly, we also observed that in the Treatment I condition, the reward-receiving cuttlefish spent significantly less time making decisions in the 1 vs*.* 0.75 test (Fig. 2b), where the choice outcome was still a preference for the larger shrimp (Fig. 3b). This finding seems to deviate from the assumption linked to the classical judgment bias test. It therefore suggests that factors other than the emotion-like state may be contributing to the observed shortened reaction latency of the cuttlefish with the unexpected reward in a difficult test situation.

In the Experiment 2, it is evident that the cuttlefish fed with a tiny shrimp before test would speed up their decision time regardless the level of discrimination tests (Fig. 4). This result demonstrates that no matter the rewards were unexpected or expected, once cuttlefish had the shrimp before test, their latency of making choice between different sizes of prey would decrease. From a metabolic point of view, the reward itself may elevate the appetite of the cuttlefish, and this could lead to an increased tendency to try to obtain the food, which in turn would lead to a shorter reaction latency when making decisions during three different levels of discrimination tests. It has been suggested in rodents that consuming food rewards might induce feelings of “wanting” and “liking” via two separate pathways^53,54^. Incentive salience or “wanting” is behaviorally expressed as changes in transitions of goal-directed behavioral patterns, which are labeled as anticipatory or appetitive behaviors^55^. Accumulated evidence now suggests that dopamine is critical to motivating animals to seek or want rewards during goal-directed behavior^56^. On the other hand, the actual pleasurable impact of reward consumption, “liking”, is mediated by only small subregions of these greater mesolimbic structures. The “liking” system has the ability to elicit affective facial expressions of “liking” when neurochemically stimulated by opioid and endocannabinoid neurotransmitters, but not dopamine^57^. Evidence for the existence of distinct neural pathways governing “liking” and “wanting” in rodents perhaps implies that the pre-test reward in the present study caused incentive salience rather than sensory pleasure in cuttlefish.

In the Experiment 3, even the tentacle strike alone without actually fed with the shrimp before test, cuttlefish showed a significant reduction of latency in making the choice of different prey size in the difficult and ambiguous tests (Fig. 5). This finding suggests that it is not the consumption of shrimps that affects cuttlefish’s appetite or internal state, rather the attempt of capturing the prey, regardless success or failure, is sufficient to alter cuttlefish’s preying strategy. In other words, cuttlefish can change their foraging strategy adaptively depending on their pre-test conditions. Without the rewards or other pre-test experiences, cuttlefish in a typical scenario (the Control condition) might adopt a conservative foraging strategy during which they spent more time identifying the shrimp that is relatively larger among two potential prey of similar size. However, after obtaining the pre-test reward as capital, cuttlefish have more room to bear the risk of choosing a relatively smaller shrimp from two prey of similar size. Thus, the cuttlefish’s default preference for choosing larger shrimp was reduced, and this allows cuttlefish to make a fast decision in prey size selection. Importantly, when we examined the choice outcomes of cuttlefish in three different treatments, it is evident that only the subject with the unexpected rewards showed both the speed of decision-making and the default preference of larger shrimps (Fig. 6). This observation indicates that the unexpected rewards may influence cuttlefish’s internal state and make them more speedily as well as accurately in decision-making. In one previous study using rats, it has been shown that cognitive judgment bias might interact with risk-based decision making^58^; specifically, it was found that optimistic judgment bias is associated with an increased propensity to make a risky choice. This was particularly the case when the risk level was high. In a similar manner, our findings suggest that the unexpected reward may induce cuttlefish to use a risky foraging strategy while maintain the decision speed and accuracy. In fact, cuttlefish are opportunistic predators in the wild^59^. Nevertheless, they choose different foraging strategies based on their situations. Previous studies have shown that cuttlefish adopted selective foraging behavior in response to what the availability of their preferred prey will be in future^60^. Moreover, another study has demonstrated that the cuttlefish prefer a large shrimp over two smaller ones when they have been starved, but chose the two small ones when they have been fed to satiation. This suggests that cuttlefish are making foraging decisions based on a state-dependent valuation^43^. Yet in another study, it has also been shown that forage decision making by cuttlefish is dependent on relative values learned during previous experiences^44^. In the present study, we demonstrate that the unexpected reward was able to affect risk assessment by cuttlefish and this consequently changes their foraging strategy.

Our original aim was to investigate whether cuttlefish were able to experience an internal emotion-like state by using a behavioral paradigm inspired by the judgment bias test. Hence, our methodology was significantly deviated from the classis judgment bias test because (1) there was no previous training or conditioning with different stimuli, and (2) the discriminative test was conducted in parallel rather than in succession. Taken all together, this could suggest that this behavioral paradigm is not appropriate to investigate cuttlefish’s emotion-like state. Indeed, without a proper training, the response to the ambiguous stimuli cannot be associated and subsequently interpreted in relation to the trained stimuli. Future research into the possibility of emotion-like state in cephalopods may adopt alternative methods, such as attentional bias^61^ or anticipatory behavior^55^, to assess their affective states.

In summary, our findings indicate that pre-test treatments of unexpected and expected rewards or simply unsuccessful visual attack could induce cuttlefish to change their foraging behaviors. The fact that the choice outcomes of cuttlefish with different pre-test experiences in three levels of discrimination tests were different suggests that multiple factors could work together to shape the cuttlefish’s choice behavior. This context dependent decision making implies that cuttlefish’s foraging strategies are influenced by the previously surprised event and their internal states.

## Supplementary Information

Supplementary Information.

## Data Availability

Data available in the electronic supplementary material.
